# Video Evidence of Tissue Sliding Improvement by Ultrasound-Guided Hydrorelease on Scars After Arthroscopic Knee Surgery: A Case Report

**DOI:** 10.7759/cureus.27975

**Published:** 2022-08-13

**Authors:** Takahiro Machida, Michiko Fukao, Akihisa Watanabe, Shinichi Miyazawa

**Affiliations:** 1 Orthopaedics, Machida Orthopaedics, Kochi, JPN; 2 Rehabilitation, Machida Orthopaedics, Kochi, JPN; 3 Orthopaedic Surgery, National Hospital Organization, Fukuyama Medical Center, Fukuyama, JPN

**Keywords:** dynamic ultrasonography, video evidence, ultrasound-guided hydrorelease, postoperative scarring, anterior knee pain, after arthroscopy

## Abstract

Postoperative scarring is a complication of arthroscopic knee surgery that causes a lack of terminal extension and tissue sliding defects. We present video evidence of tissue sliding before and after ultrasound-guided hydrorelease in a 53-year-old man. The patient presented with pain in the scarred area following arthroscopic knee surgery. His active and passive extension was -5° with restricted patellar mobility. Dynamic ultrasonography revealed scar tissue sliding defects. For ultrasound-guided hydrorelease, a needle (22G, 60 mm) was aimed at a site within 10 mm depth of the hypoechoic change in the scar area below the patella, and saline solution (10 mL) mixed with 1% lidocaine (10 mL) and 10 mg prednisolone was injected. Immediately after injection, the patient's extension was 0° with no pain or limitation of patellar mobility, and dynamic ultrasonography showed tissue sliding improved. Video evidence from dynamic ultrasonography clarifies the direction of the inadequate slide and the indication for and efficacy of ultrasound-guided hydrorelease. This case highlights the benefits of video evidence from dynamic ultrasonography before and after ultrasound-guided hydrorelease.

## Introduction

Postoperative scarring is a complication of arthroscopic knee surgery and contributes to progressive fibrosis in the infrapatellar fat pad, lack of terminal extension, anterior knee pain (AKP), and decreased patellar mobility [1,2]. The proposed treatments for this issue are anterior interval release [3,4] and manual therapy [5], although multiple arthroscopic surgeries may lead to additional complications. A less invasive treatment than arthroscopic surgery would be beneficial for patients suffering from these types of pathologies.

Ultrasound-guided hydrorelease has been performed for lower back pain [6] and postoperative scar tissue [7]. Machida et al. consider ultrasound confirmation of improved tissue sliding as the key to the success of this procedure regarding the treatment of scar tissue after arthroscopic surgery. However, no video evidence of improved tissue sliding has been presented [7]. We performed an ultrasound-guided hydrorelease in a patient who presented with a lack of terminal extension and AKP after arthroscopic surgery for medial meniscus injury. We present video evidence of tissue sliding before and after the procedure in this patient.

## Case presentation

**Figure 1:**
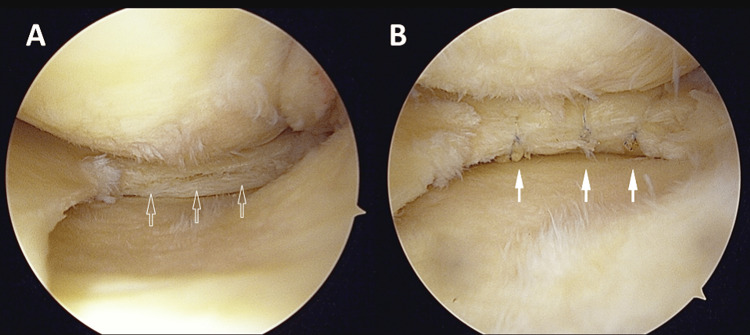
Arthroscopic views in the patient's left knee A) Before repair: Horizontal tear of the medial meniscus (arrow). B) After repair: Horizontal tear of the medial meniscus repaired by the all-inside repair technique (arrow).

A 53-year-old man (height: 170 cm; body weight: 80 kg; body mass index: 27.7 kg/m^2^), diagnosed with medial meniscus injury, presented with AKP after previous arthroscopic knee surgery. He developed left knee pain six months before surgery and underwent arthroscopic medial meniscus repair after conservative therapy failed to improve his pain. Arthroscopic surgery was performed according to the standard all-inside repair technique using FasT-Fix system (Smith & Nephew Endoscopy, Andover, USA) [8] (Figure 1), and range-of-motion exercise was started after two weeks of knee brace immobilization. However, his range of motion remained limited even at six weeks postoperatively, and the scar under the patella tightened during extension, causing him severe pain. Active and passive extension was -5° with restricted patellar mobility, passive flexion was 115°, and an 11-point numerical rating pain scale (NRPS; 0 (“no pain”) to 10 (“worst possible pain”)) was three in walking. The Knee injury and Osteoarthritis Outcome Score (KOOS) showed KOOS pain of 25.0, KOOS symptoms of 17.9, and KOOS activities of daily living (ADL) of 47.0 (Table 1) [9].

**Table 1:** The patient's range of motion and function course NRPS: Numerical rating pain scale; KOOS: The Knee injury and Osteoarthritis Outcome Score; ADL: Activities of daily living; N/A: Not available.

	Six weeks postoperative, before hydrorelease	Seven weeks postoperative, after hydrorelease	15 weeks postoperative
Knee extension, degree	-5	0	0
Knee flexion, degree	115	120	145
Pain in walking, NRPS	3	1	1
KOOS pain	25	N/A	66.7
KOOS symptoms	17.9	N/A	57.1
KOOS ADL	47	N/A	91.0

The dynamic ultrasonography six weeks postoperatively revealed scar sliding defects during quadriceps contraction (Video 1) and manual skin sliding (Video 2). We then performed ultrasound-guided hydrorelease, as reported by Machida et al. [7].

**Video 1:** Dynamic ultrasonography at medial scar area during quadriceps contraction A) Before ultrasound-guided hydrorelease: The hypoechoic area is present in the scar area. Tension from quadriceps contraction is not transmitted distally (arrow). B) After ultrasound-guided hydrorelease: Tension from quadriceps contraction is transmitted distally beyond the hypoechoic area of the scar (arrow).

**Video 2:** Dynamic ultrasonography at medial scar area during manual skin surface sliding A) Before ultrasound-guided hydrorelease: Manually sliding over the skin surface does not slide over the hypoechoic areas of the scar (arrow). B) After ultrasound-guided hydrorelease: The movement of the scar area during manual sliding on the skin surface is greater than before ultrasound-guided hydrorelease (arrow).

For the ultrasound-guided hydrorelease, the patient was positioned in 30° flexion of the knee joint in the supine position. Ultrasonography was performed using a three to 11 MHz B-mode linear array scanner (SONIMAGE MX1, Konica Minolta, Tokyo, Japan). For ultrasound-guided hydrorelease, a needle (22G, 60 mm) was aimed at a site within 10 mm depth of the hypoechoic change in the scar area below the patella, and saline solution (10 mL) mixed with 1% lidocaine (10 mL) and 10 mg prednisolone was injected: first on the lateral scar below the patella and then the same for the medial scar the following week, and Video 1 and Video 2 show the medial scar. Only one injection was performed for each, and the treatment time was approximately 10 minutes, including an ultrasound examination. All procedures were performed by one orthopedic surgeon with more than 10 years of experience.

Immediately after second injection, left knee extension was 0° without pain, flexion was 120°, and pain in walking was a score of one on the NRPS. Dynamic ultrasonography showed that scar tissue sliding improved (Video 1 and 2). After that, rehabilitation continued with a range of motion exercise, manual therapy [5], mobilization of the patella, and muscle strengthening. At 15 weeks postoperatively (i.e., at nine weeks post hydrorelease), his left knee extension was 0° with no pain or limitation of patellar mobility, flexion was 145°, and pain in walking was one on NRPS. KOOS pain, symptoms, and ADL scores were 66.7, 57.1, and 91.0, respectively. After the injections, the patient felt heaviness in the left knee for one day, which improved spontaneously without any specific treatment. After that, the heaviness never recurred. He was satisfied with the ultrasound-guided hydrorelease and subsequent rehabilitation and could return to work as an auto mechanic.

## Discussion

The most important finding from this patient's course was that ultrasound-guided hydrorelease to the scar after previous arthroscopic knee surgery improved the terminal extension with AKP. Furthermore, the video evidence showed that scar tissue sliding had indeed improved.

The patient's terminal extension was ultimately improved by ultrasound-guided hydrorelease. A previous case series on arthroscopic anterior interval release showed that all patients with a lack of terminal extension had improved [10]. We believe ultrasound-guided hydrorelease may also be effective, as this patient's progress demonstrates. The advantage of this procedure is that it is less invasive than arthroscopic anterior interval release, which may be beneficial for postoperative patients.

Notably, the video evidence objectively confirmed that scar tissue sliding improved. This highlights the effectiveness of dynamic ultrasonography. The video evidence also identifies the stimulus and direction of the sliding defects. With video evidence, we can clearly visualize the indications and results of ultrasound-guided hydrorelease. Evaluation of the anterior interval scar is recommended for patients suffering from lack of terminal knee extension, AKP, and decreased patellar mobility, which may be indications for arthroscopic release [4]. We recommend that surgeons confirm the scars in patients presenting with similar symptoms and attempt dynamic ultrasonography and ultrasound-guided hydrorelease before arthroscopic anterior interval release.

In a previous report by Machida et al. [7], who proposed this technique, the mechanism of tissue separation by ultrasound-guided hydrorelease may have improved tissue sliding. Therefore, confirmation of the sliding by ultrasonography will be the key to the success of this procedure. To the best of our knowledge, this is the first report to present video evidence of improved tissue sliding before and after ultrasound-guided hydrorelease for arthroscopic scars.

Ultrasound-guided hydrorelease has several strengths. It can immediately improve terminal knee extension in patients with AKP. Also, because of its less invasive procedure, knee brace immobilization or unweighting are not necessary, and exercise therapy can begin immediately. Another major strength is that the procedure is safe. The patient had no serious adverse events requiring specific treatment. Furthermore, no serious adverse events have been reported in previous studies [6,7,11]. Finally, ultrasound-guided hydrorelease can be presented on videos, which can aid in informing the patient before obtaining the patient's consent for the procedure.

Despite these benefits, there are still some unknowns with ultrasound-guided hydrorelease. First, the timing of its application is unknown. In a previous study by Machida et al. [7], the patient was in the tenth month after arthroscopic knee surgery, whereas in this case report, the patient was in the sixth week after surgery. The second is an injection agent. We injected saline mixed with lidocaine and prednisolone because the patient felt severe pain. In previous studies, saline only [6,7], saline mixed with 0.5% mepivacaine hydrochloride [11], and bicarbonate Ringer's solution [11] have been used. In a comparison of the analgesic effects of injectable agents, saline alone has been reported to be more analgesic than local anesthesia. [12].

## Conclusions

Ultrasound-guided hydrorelease applied to the scar after arthroscopic surgery resulted in full extension of this patient's knee joint without pain and video evidence of improved sliding was presented. Video evidence from dynamic ultrasonography has the advantage of clarifying the direction of the lack of sliding and the indication for and efficacy of ultrasound-guided hydrorelease. This case highlights the benefits of video evidence from dynamic ultrasonography before and after ultrasound-guided hydrorelease.

## References

[1] Ahmad CS, Kwak SD, Ateshian GA, Warden WH, Steadman JR, Mow VC (1998). Effects of patellar tendon adhesion to the anterior tibia on knee mechanics. Am J Sports Med.

[2] Murakami S, Muneta T, Ezura Y, Furuya K, Yamamoto H (1997). Quantitative analysis of synovial fibrosis in the infrapatellar fat pad before and after anterior cruciate ligament reconstruction. Am J Sports Med.

[3] Rooney A, Wahba AJ, Smith TO, Donell ST (2015). The surgical treatment of anterior knee pain due to infrapatellar fat pad pathology: a systematic review. Orthop Traumatol Surg Res.

[4] Rose M, McNeilan R, Genuario J, Schlegel T (2018). Surgical technique for release of anterior interval scarring of the knee after anterior cruciate ligament reconstruction. Arthrosc Tech.

[5] Alvira-Lechuz J, Espiau MR, Alvira-Lechuz E (2017). Treatment of the scar after arthroscopic surgery on a knee. J Bodyw Mov Ther.

[6] Kanamoto H, Orita S, Inage K, Shiga Y, Abe K, Eguchi Y, Ohtori S (2021). Effect of ultrasound-guided hydrorelease of the multifidus muscle on acute low back pain. J Ultrasound Med.

[7] Machida T, Watanabe A, Miyazawa S (2020). A new procedure for ultrasound-guided hydrorelease for the scarring after arthroscopic knee surgery. Cureus.

[8] Li WP, Chen Z, Song B, Yang R, Tan W (2015). The FasT-Fix repair technique for ramp lesion of the medial meniscus. Knee Surg Relat Res.

[9] Roos EM, Roos HP, Lohmander LS, Ekdahl C, Beynnon BD (1998). Knee Injury and Osteoarthritis Outcome Score (KOOS)—development of a self-administered outcome measure. J Orthop Sports Phys Ther.

[10] Steadman JR, Dragoo JL, Hines SL, Briggs KK (2008). Arthroscopic release for symptomatic scarring of the anterior interval of the knee. Am J Sports Med.

[11] Kobayashi T, Kimura H, Ozaki N (2016). Effects of interfascial injection of bicarbonated Ringer’s solution, physiological saline and local anesthetic under ultrasonography for myofascial pain syndrome - two prospective, randomized double-blinded trials. J Juzen Med Soc.

[12] Frost FA, Jessen B, Siggaard-Andersen J (1980). A control, double-blind comparison of mepivacaine injection versus saline injection for myofascial pain. Lancet.

